# Quantifying the Dietary Overlap of Two Co‐Occurring Mammal Species Using DNA Metabarcoding to Assess Potential Competition

**DOI:** 10.1002/ece3.71274

**Published:** 2025-04-11

**Authors:** Aurelie M. Kanishka, Christopher MacGregor, Linda E. Neaves, Maldwyn John Evans, Natasha M. Robinson, Nick Dexter, Chris R. Dickman, David B. Lindenmayer

**Affiliations:** ^1^ Fenner School of Environment and Society The Australian National University Canberra Australian Capital Territory Australia; ^2^ Conservation and Restoration Science Branch, Science, Economics and Insights Division NSW Department of Climate Change, Energy, the Environment and Water Parramatta New South Wales Australia; ^3^ Booderee National Park Jervis Bay Australian Capital Territory Australia; ^4^ School of Life and Environmental Sciences The University of Sydney Sydney New South Wales Australia

**Keywords:** bush rat, common brushtail possum, competition, dietary generalist, DNA metabarcoding

## Abstract

Interspecific competition is often assumed in ecosystems where co‐occurring species have similar resource requirements. The potential for competition can be investigated by measuring the dietary overlap of putative competitor species. The degree of potential competition between generalist species has often received less research attention than competition between specialist species. We examined dietary overlap between two naturally co‐occurring dietary generalist species: the common brushtail possum *Trichosurus vulpecula* and the bush rat *Rattus fuscipes* . To gauge the potential for competition, we conducted a diet analysis using DNA extracted from faecal samples to identify the range of food items consumed by both species within a shared ecosystem and quantify their dietary overlap. We used DNA metabarcoding on faecal samples to extract plant, fungal, and invertebrate DNA, identifying diet items and quantifying dietary range and overlap. The species' diets were similar, with a Pianka's overlap index score of 0.84 indicating high dietary similarity. Bush rats had a large dietary range, consisting of many plant and fungal species and some invertebrates, with almost no within‐species variation. Possums had a more restricted dietary range, consisting primarily of plants. We suggest that the larger dietary range of the bush rat helps buffer it from the impacts of competition from possums by providing access to more food types. We conclude that, despite the high ostensible overlap in the foods consumed by dietary generalist species, fine‐scale partitioning of food resources may be a key mechanism to alleviate competition and permit co‐existence.

## Graphical abstract

Interspecific competition among co‐occurring species with similar resource requirements can be assessed by measuring diet overlap. A study on common brushtail possums and bush rats used DNA metabarcoding of faecal samples to quantify their dietary overlap, finding a significant similarity. Despite this overlap, the bush rat's broader dietary range and differences in diet among its population components likely mitigate competition, allowing both species to coexist through fine‐scale resource partitioning.

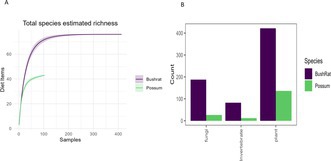

## 1 Introduction

Food resources are vital for animal growth, survival, and reproduction. However, the availability of these resources, especially when in short supply, can influence the behaviours and decision‐making of foragers and their interactions with other individuals (Corman et al. 2016). Scarce food resources can result in species shifting when and where they forage, what they forage on, diet switching and adapting their foraging behaviours (Chard et al. 2017; Favreau et al. 2018). Group‐living animals such as grey kangaroos *Macropus giganteus* often forage in smaller groups during periods of low food availability and adjust their feeding behaviours, such as by increasing vigilance, to reduce risks of predation (Favreau et al. 2018). In other group‐living species, such as some seabirds, high population sizes can induce individuals to travel further to forage to reduce the potential for intraspecific competition (Corman et al. 2016; Lamb et al. 2017). Food availability can also influence the choice of food types, as has been shown with Australian white ibis *Threskiornis molucca* (Chard et al. 2017) and Black‐footed titi *Cellicebus nigrifrons* (Nagy‐Reis and Setz 2017).

Although food availability and quality often determine the foraging decisions of individuals within a population, this can also have powerful effects on individuals of different species if these taxa have overlapping niches and compete for food (Bell et al. 2023; Kumpan et al. 2019; Santosa et al. 2012). For example, Santosa et al. (2012) found that white‐bearded gibbons *Hylobates albibarbis* and leaf monkeys *Presbytis rubicunda* in west Kalimantan shifted their foraging patch use in response to the availability of food, avoiding each other when food was scarce and being most likely to share foraging patches when fruit availability was high. Furthermore, Bell et al. (2023) showed that foraging patterns in the presence of competition can have long‐term influences on the activity and distribution of multiple interacting species within ecological communities, with subordinate foragers exhibiting spatial and temporal avoidance of dominant species.

The intensity and effects of interspecific dietary competition can change with fluctuating availability of shared food resources (Prevedello et al. 2013). Resources can become limited through the increased abundance of one or more competitor species (Dexter et al. 2013; Treloar et al. 2021). According to Treloar et al. (2021), as the population density of one species increases, the availability of shared resources for heterospecific individuals decreases, which can lead to more intense competition and declines of subordinate species. Understanding how species share resources at different levels of resource availability can be vital for predicting when competition becomes particularly detrimental for one or more of the competitors, especially when accounting for changing environmental conditions (Gebremedhin et al. 2016).

Trophic, or dietary, generalists are often viewed as being more resilient to environmental change than their specialist counterparts as they can switch to alternative food if components of their diet become scarce (Lloyd and Vetter 2019; Treloar et al. 2021). For example, the boodie *Bettongia lesueur* , in Western Australia, is characterised by behavioural plasticity and a broad dietary niche, allowing the species to adapt readily to new environments when it is moved in conservation translocations (Bice and Moseby 2008; Treloar et al. 2021). However, dietary generalist species are usually also considered to be weaker competitors when compared to specialists, as specialists tend to be highly efficient at competing for a restricted set of resources, whereas generalists will switch their use of resources in response to resource limitation (Barnes and Murphy 2023; de Carvalho et al. 2019; Püttker et al. 2019). This can result in generalist species having patchy or ‘checkerboard’ distributions within communities of specialists or thriving primarily in environments where specialists are absent (Nordberg and Schwarzkopf 2019; Püttker et al. 2019). Where dietary generalist species do co‐occur with specialists, they will often segregate spatially or temporally, or in their diet, to avoid aggressive competitive interactions (Kent et al. 2022). Conversely, generalist‐generalist interactions have rarely been a focus of research (Barnes and Murphy 2023), despite theoretical interest and expectations that high overlap along much of the food resource axis between generalist species should increase the likelihood of competition between them (Dickman 2011). However, the amount of specialisation in diet that species display is often affected by seasonal changes and variations in resource limitations (Kent et al. 2022). This in turn, can influence foraging behaviour and competition, resulting in an increase in the occurrence of generalist–generalist competition (Kent et al. 2022).

Although competition between species can be confirmed only in carefully controlled removal or addition experiments in which scarce resources are identified (Dickman 2011), a common first step in clarifying the potential for competitive interactions is to measure the extent of niche overlap between putative competitor species (Villsen et al. 2022). If food is suspected to be a scarce resource, the species' diets need to be described and compared (Nordberg and Schwarzkopf 2019; Wang et al. 2023). However, this can be a difficult task for dietary generalist species owing to the wide range of food types that may be consumed (Calver and Loneragan 2024). High dietary breadth at the population level can arise if all animals consume a similarly wide range of foods, or if individuals specialise in different food types (Van Valen 1965). In the latter case, consumers may exhibit age, sex, habitat or even personality‐related differences in their diets, with the population viewed collectively as having a generalist diet (Bolnick et al. 2003; Dickman and Newsome 2015; Woo et al. 2008). The study we report here focused on the diets of the bush rat *Rattus fuscipes* , a native Australian rodent (~100 g), and the common brushtail possum *Trichosurus vulpecula* (hereafter ‘possum’, ~2 kg), a partly arboreal marsupial, within Booderee National Park (BNP) in south‐eastern Australia. Both species are considered to be generalist omnivores (Callander 2018; Cruz et al. 2012) that consume similar ranges of plants, fungi, invertebrates, and other food types, although dietary overlap has not been assessed in any sites where the two species co‐occur. Recent studies of these species within BNP have demonstrated that, for several years, possums have increased in abundance, while bush rats have decreased (Kanishka et al. 2023; Lindenmayer et al. 2018). The decline in bush rats may be a response to the increase in the number of possums, which are considerably larger and potentially competitively dominant (Kanishka et al. 2023, 2025). The two species are taxonomically distinct and differ in behaviour and habitat use, with bush rats being largely ground‐dwelling and exhibiting a preference for dense, complex vegetation, and possums being partly arboreal (Callander 2018; Cruz et al. 2012). However, possums also spend significant periods foraging on the ground and in the understorey (Cruz et al. 2012), increasing both the chance of encounter between species and the number and range of resources they share. A recent study has indicated that bush rats will reduce foraging time and effort in sites shared with possums, potentially to avoid detrimental competition (Kanishka et al. 2025). We hypothesised that based on trophic niche partitioning theories (Villsen et al. 2022), as possum abundance increases, the time they spend on the ground will increase, leading to an increase in competition for shared food resources. We further hypothesised this will have detrimental effects on the smaller and putatively subordinate bush rat (Dexter et al. 2013; Treloar et al. 2021).

Using DNA metabarcoding, we aimed to characterise the diets of bush rats and common brushtail possums at both a broad overall level and at a finer scale, characterising within‐species variation by calculating their niche breadths and dietary ranges. DNA metabarcoding utilises the degraded DNA in faecal samples to identify the taxa likely to have been consumed, and can provide greater sensitivity compared to histological and morphological methods (Alemany et al. 2023; Villsen et al. 2022). We also aimed to determine the level of overlap in their diets at multiple scales. We anticipated that the degree of dietary overlap would provide an indication of the potential for competition between the two species, and potentially uncover a mechanism explaining their inverse numerical relationship in Booderee National Park. We predicted that: (1) the diets of common brushtail possums and bush rats would encompass a broad range of food types, (2) there would be a high overlap in the range of food types consumed by the two species, and (3) fine‐scale differences in diet would occur within and between species with respect to sex, age, and vegetation type where animals were active.

## 2 Methods

### 2.1 Ethics Statement

This study was conducted in strict accordance with the recommendations in the *Australian Code for the Care and Use of Animals for Scientific Purposes*. The protocol was approved by the Animal Experimentation Ethics Committee at the Australian National University (Protocol Number: A2020/25).

### 2.2 Study Area

**FIGURE 1:**
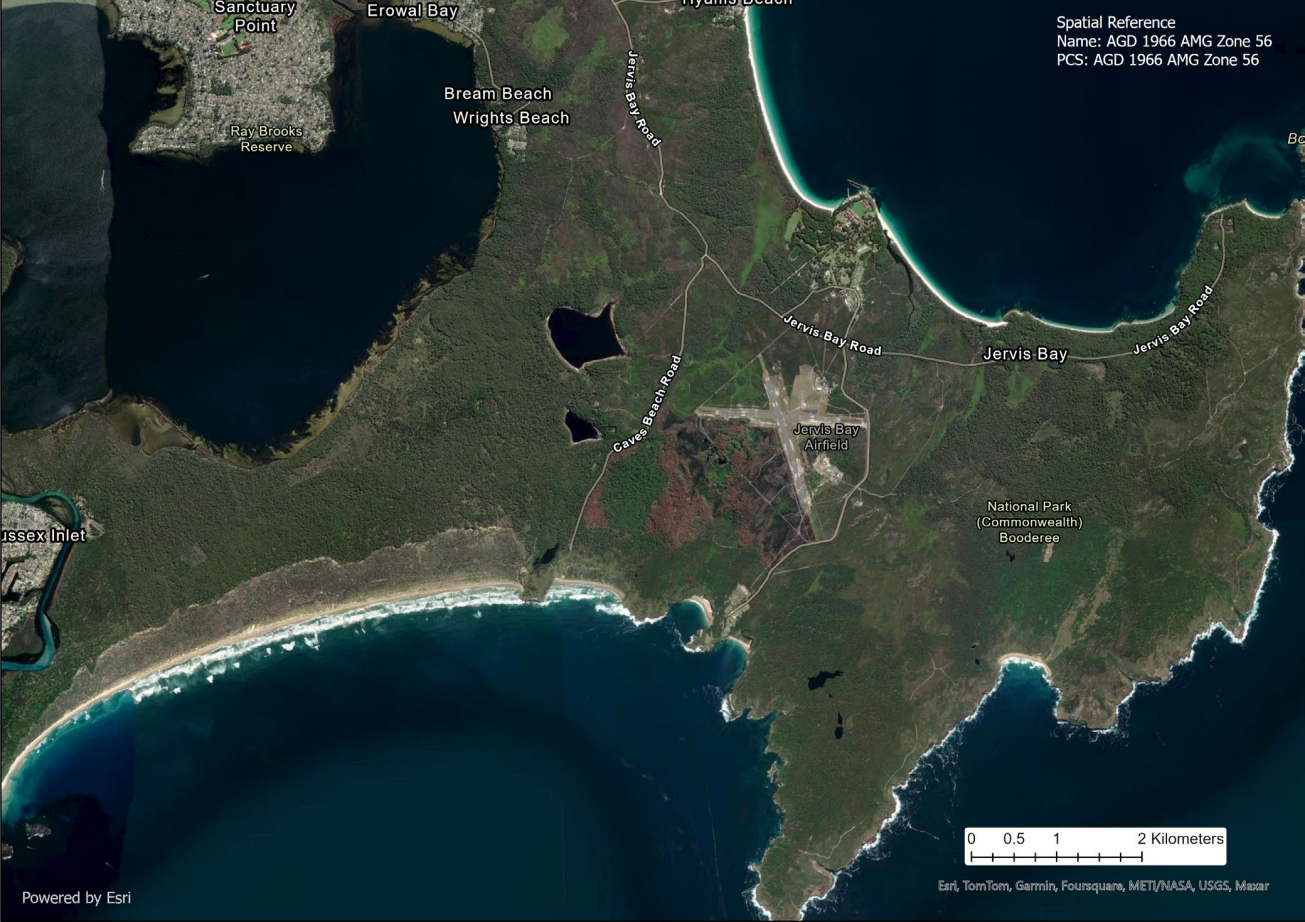
Satellite map of Booderee National Park, Jervis Bay Territory, Australia (Esri, 2022). All green, vegetated areas are within the boundary of BNP.

We conducted our study at Booderee National Park (BNP), Jervis Bay Territory, Australia (Figure 1). Booderee National Park is co‐managed by the Wreck Bay Aboriginal Community and Parks Australia. It has a temperate climate (BOM 2021). The Park has undergone an intensive program of poison baiting to reduce the numbers of the introduced red fox *Vulpes vulpes* since 2002 (Dexter et al. 2013; Lindenmayer et al. 2005, 2018). The reduction in predation pressure from the red fox has potentially allowed the possum population to increase (Lindenmayer et al. 2018). The remains of both possums and bush rats occur frequently in the diets of red foxes in coastal forest areas (Fleming et al. 2021). However, the removal of foxes by poison baiting has much stronger positive effects on possums than on bush rats (Banks 1999; Dexter and Murray 2009; Kovacs et al. 2012).

### 2.3 Faecal Collection

We collected samples over two trapping periods: the first in October 2020 (40 samples) and the second between December 2021 and February 2022 (95 samples). These comprised 57 possum and 78 bush rat faecal samples. We collected samples fresh from cage traps and Elliott traps that had been set to capture the two species after the animals had been processed and released on the morning following capture (Lindenmayer et al. 2008; Macgregor et al. 2020). We stored the samples collected in the first period in ethanol in 5 mL tubes. We stored the samples collected in the second period with silica gel in 15 mL tubes at an approximate ratio of 1:4 faecal sample to silica gel. We stored all samples at −20°C prior to DNA extraction. We also recorded the sex and approximate life stage (hereafter referred to as ‘age’) of all trapped animals, as well as the location and broad vegetation type (forest, woodland, rainforest, sedge, heath, shrub, and casuarina) where the samples were collected from (Table S1) (Taws 1998). We estimated age using visual information on body size and development of the external reproductive organs, distinguishing animals as juvenile, subadult or adults.

### 2.4 DNA Extraction

We extracted DNA from the faecal samples using the DNeasy PowerSoil Pro Kit (Qiagen, Hilden, Germany). To improve the amplification process, we cleaned the samples in several rounds, first with the DNeasy PowerClean Pro clean‐up kit (Qiagen, Hilden, Germany), and second using Spri‐Speed magnetic beads (Beckman Coulter, New South Wales, Australia). Extraction controls were used to check for any contamination.

We amplified each sample with primers to extract plant, fungal, or invertebrate DNA separately with polymerase chain reactions (PCRs). The primer we used for plant DNA was the chloroplast *trn*L intron (referred to as *trn*L) (Taberlet et al. 2007). The primer we used for invertebrate DNA was the mitochondrial cytochrome oxidase subunit I sequence (referred to as COI) (Shutt et al. 2021). Negative PCR controls were included in all reactions. The primer we used for fungal DNA was the ITS3/ITS4 primer, which targets the 18S rRNA gene and Internal Transcribed Spacer region (referred to as ITS) (Nuske et al. 2018).

We indexed the amplified DNA using the NEBNext Multiplex Oligos dual index primers (New England Biolabs, Massachusetts, USA). We further cleaned the indexed amplified DNA with Sera‐Mag SpeedBeads (Cytiva, Victoria Australia) and pooled the amplicons in equimolar volumes. We constructed a library using the V2 2 × 250 base pair kit (Illumina Inc., California, USA). Finally, we sequenced the samples using paired‐end sequencing on an Illumina MiSeq sequencer (Illumina Inc., California, USA).

### 2.5 Data Analysis

We analysed all sequences using the dada2 ver. 1.16.0 (Callahan et al. 2016), Biostrings ver. 2.60.2 (Pagès et al. 2023) and Cutadapt ver. 4.7 (Martin 2011) packages in R ver. 4.4.1 (R Core Team 2021). Using the Biostrings and Cutadapt packages, we trimmed all sequences of the primer and adaptor sequences. We also used the dada2 package to filter sequences, check for error rates, and remove chimeric sequences.

We created three reference libraries, one for each taxon set. For the plant taxa, we used a plant list for BNP (collected by C. Foster) and collected the relevant sequences from NCBI Genbank (NCBI 2023). We then checked these sequences using the AliView program (Larsson 2014). For the invertebrate taxa, we collated sequence records from Canberra, Sydney, and Nowra, NSW (a town close to BNP) from the BOLD database (BOLD Systems 2023). For the fungal taxa, we collected sequence records from Australia from the UNITE database (downloaded November 2022) (Nielsen et al. 2017). We created a subset of ectomycorrhizal fungi from the fungal records for subsequent analyses, to focus on fungi most likely to be actively consumed. We then assigned our sequences to taxa from the reference libraries using the ‘assignTaxonomy’ function in the dada2 package, which uses a Bayesian likelihood method to assign sequences to the most likely taxa, or the best fitting higher order taxa (i.e., order, family) (Callahan et al. 2016). For sequences that were not assigned during the Bayesian process and had been detected in the barcodes for five or more individuals of either species, we manually searched for these sequences in the NCBI nucleotide database (NCBI 2023), using the BLASTn algorithm system (Altschul et al. 1990).

Of the resulting data, we dropped sequences with less than 10 reads for an individual (Mardis 2008). We then converted the reads to binomial data (i.e., 0 for < 10 reads, identified as not present in the diet, and 1 for ≥ 10 reads, identified as present in the diet). Finally, we converted the sequences to their lowest assigned taxonomic level. We completed all statistical analyses at the lowest available taxonomic level, while diets were visually compared at the ordinal level (Figures 3, 4, 5).

### 2.6 Statistical Analysis

We used two methods to analyse and characterise the dietary niche range of bush rats and possums. First, we analysed the frequency of items within bush rat and possum diets (i.e., the species' faecal samples) using one‐sample Chi‐squared tests in R (R Core Team 2021). This analysis informed us if there was an even or uneven spread in the consumption of the dietary items and allowed us to identify and investigate diet items that were consumed more frequently (Pearson 1900; Zarkami et al. 2020). We additionally used Chi‐squared analysis to investigate within‐species variation in diets. The within‐species variables we used were the sex and age of individuals and the broad vegetation type in which we trapped animals.

Second, we employed the ‘niche.width’ function from the spaa package ver. 0.0.2 (Zhang 2016) to calculate the niche breadth of each species using Levins' measure of niche breadth (Levins 1968). The Levins' measure calculates the uniformity of the distribution of resources used by individuals (Krebs 2014; Levins 1968). The higher the value of Levins' measure, the broader the dietary range of the species (Krebs 2014; Levins 1968). To compare the diet ranges based on Levins' measures between species, we compared the difference in Levins' measures to a permuted dataset, with 10,000 permutations, to calculate a predicted *p*‐value of the difference in scores, while taking the differences in sample sizes into account, by randomising the Levins' measure of the categories and calculating a probability that significant differences would occur. We calculated Levins' measures for each species and for each within‐species category.

We used two methods to calculate the dietary overlap between the study species. First, we used the ‘niche.Overlap’ function from the spaa package ver. 0.0.2 (Zhang 2016) to calculate the Pianka index of similarity (Pianka 1972; Wang et al. 2023). The Pianka index calculates a proportion of overlap between two categories based on the distribution of resources used by each category (Pianka 1972; Wang et al. 2023).

Second, we performed a PERMANOVA using Jaccard's measure of similarity using the vegan package ver. 2.6–4, to quantify dissimilarity in the diets of bush rats and possums (Oksanen et al. 2022). We first tested the homogeneity of group dispersions using the ‘betadisper’ function to determine if the data were dispersed normally, ensuring the sample size would not bias the results (Oksanen et al. 2022). We calculated a dissimilarity matrix between the dietary content of the individuals by calculating a Jaccard similarity matrix using the ‘vegdist’ function (Oksanen et al. 2022). Jaccard's similarity index measures the proportion of the number of samples in common between datasets (Kefford et al. 2010). We then compared matrices between the species using the ‘adonis2’ function (Oksanen et al. 2022) to calculate a PERMANOVA, which assesses differences between groups (Anderson 2017). We included the age, sex, and habitat type of the samples as covariates.

## 3 Results

### 3.1 Overall Niche Use

**FIGURE 2:**
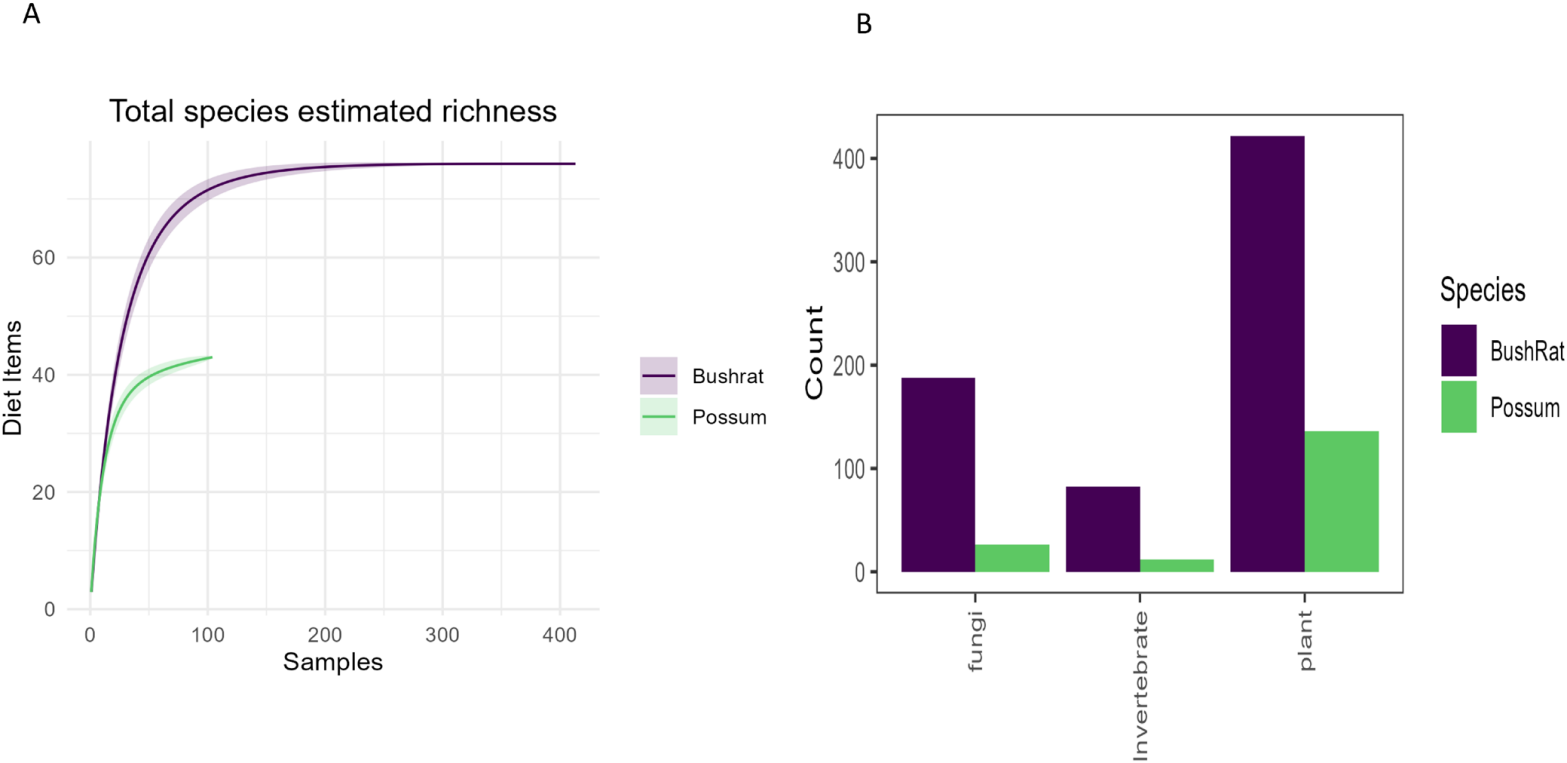
(A) Species accumulation curve of the accumulated number of diet items for the number of samples for either bush rats or possums, clearly demonstrating that bush rats both have a much greater number of samples and identified diet items (B) Numbers of diet items (i.e., counts) within each major taxon group, which were found in faecal samples of bush rats and common brushtail possums.

Between the two species, we identified 150 different dietary items to the lowest taxonomic level. Within this, bush rats consumed the wider range across a larger sample set compared to possums (Figure 2A). We detected a total of 769 sequences within the faecal samples of bush rats (*n* = 78), which we identified as belonging to 130 different taxa. Of these, 54.9% (*n* = 422) comprised plant material, 34.5% (*n* = 265) ectomycorrhizal fungi, and 10.7% (*n* = 82) comprised invertebrates. There were fewer dietary items recorded from possum faecal samples (*n* = 57), with 194 sequences detected from 39 different taxa. Of these, 70.1% (*n* = 136) comprised plant material, 23.7% (*n* = 46) were ectomycorrhizal fungi, and 6.2% (*n* = 12) were invertebrates.

Bush rats had a dietary niche breadth of 30.34, with most of their faecal samples consisting of plant material, then fungi, and only a relatively small number of invertebrates (Figure 2). There was a significant difference in the frequency with which dietary items were identified in the bush rat faecal samples, indicating that bush rats mostly consumed particular plant materials ($\chi_{84}^{2}$ = 1239.9, *p* < 0.001). Possums, relative to bush rats, had a much smaller dietary breadth of 14.25. Possum faeces, similar to bush rats, consisted primarily of plant material, followed by fungi, and almost no invertebrates (Figure 2). There was a significant difference in the frequency with which dietary items were identified in the samples, suggesting that some items were consumed more frequently than others ($\chi_{84}^{2}$ = 863.95, *p* < 0.001).

The Pianka index of overlap between the diets of bush rats and possums was 0.84. The test for dispersion demonstrated that the bush rat and possum datasets had a homogenous dispersion (*F*_1_ = 2.778, *p* = 0.098). The diets of bush rats and possums were found to be not statistically significantly different (*F*_22_ = 1.044, *p* = 0.233). The species (i.e., bush rat or possum) explained 19.3% of the variance in the data.

### 3.2 Plant Diet Items

**FIGURE 3:**
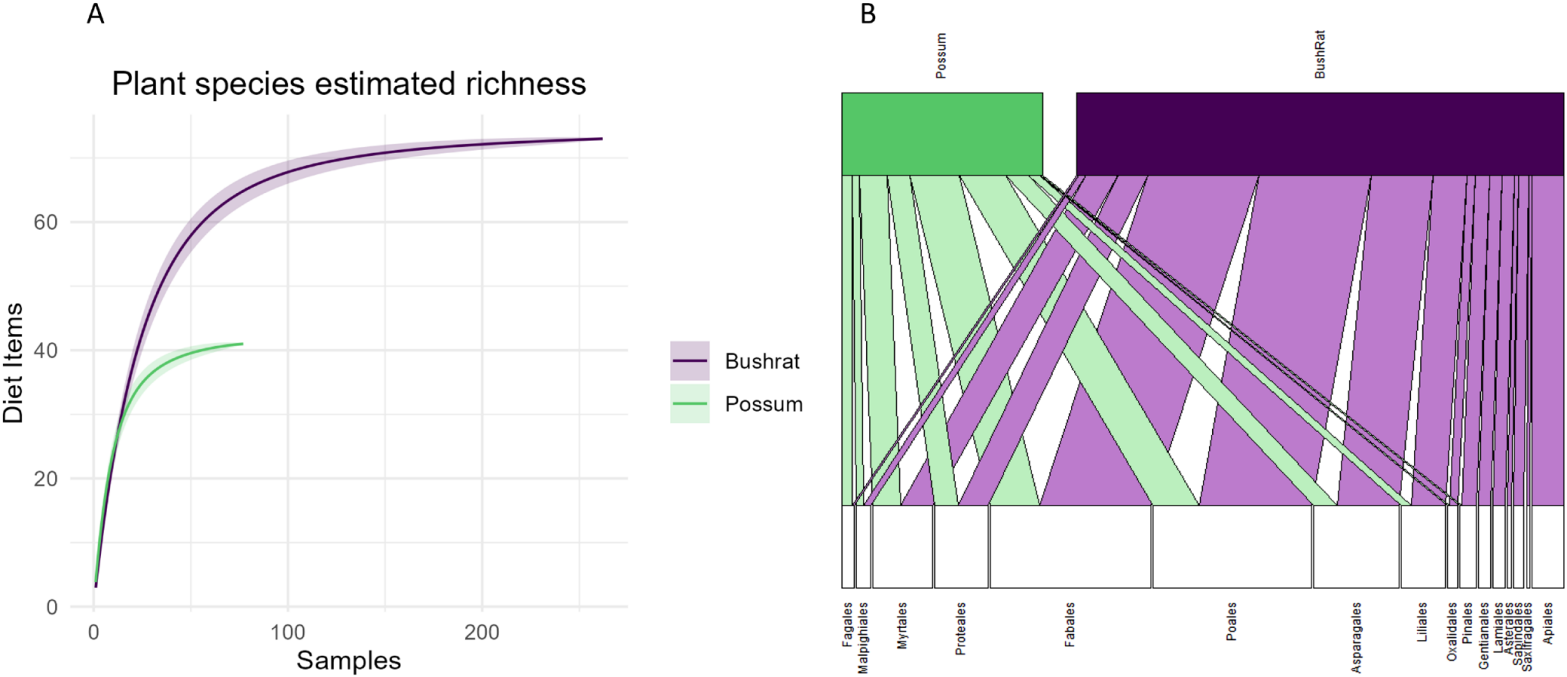
(A) Species accumulation curve of the accumulated number of diet items in the plant taxa identified for bush rats and possums for the number of samples of each species, demonstrating that bush rats had a greater species richness in dietary items for the larger sample size. (B) Bipartite network plot of the plant diet items detected in the faecal samples of bush rats and common brushtail possums at the ordinal level. This bipartite network illustrates which orders were consumed at higher frequencies, as well as illustrating where the overlaps in diets were occurring. In the plant diet, both species mostly consumed dietary items from the orders Poales and Fabales, which is also where most dietary overlap occurred.

We identified 33 dietary items within plant taxa from the faecal samples, with bush rats consuming all 33 across a larger sample set and possums consuming 19 (Figure 3A). The breadth of the species' diets was not significantly different, although bush rats had a larger dietary niche breadth (15.38) compared to possums (9.86). Both species displayed significant differences in the frequency of occurrence of different plant materials in their faecal samples (Bush rats: $\chi_{32}^{2}$ = 483.39, *p* < 0.001, Possums: $\chi_{32}^{2}$ = 319.21, *p* < 0.001). Both species consumed more plants from the order Poales (grasses and sedges) than from other plant groups. For dietary items identified to the species level, bush rats mostly consumed prickly couch grass *Zoysia macrantha* (Poaceae), and possums mostly consumed wombat berry *Eustrephus latifolius* (Asparagaceae) (Figure 3).

Within species, there was no difference in the dietary ranges of males and females for either bush rats or possums (Table 1). All groups showed that some plant materials were consumed at a higher frequency than others (Table 1). There also was no difference in the plant dietary ranges between age groups of either bush rats or possums (Table 1). Similarly, all groups consumed some plant materials more than others (Table 1). Bush rats showed similar dietary ranges between individuals in different habitat types (Table 1). There was an even frequency of consumption by bush rats in scrubland and heathland habitats, but an uneven frequency of consumption by bush rats in forest, woodland, shrubland, rainforest and sedgeland habitats. Possums did exhibit different dietary ranges between individuals in different habitat types, with possums in forest habitats having a higher dietary range (0.304), and possums in Casuarina habitats having a restricted dietary range (0.030) (Table 1). There was an uneven frequency of consumption for most possums across habitats, with evidence that mostly Poales plants were consumed; however, there was an even frequency of consumption for possums in Casuarina habitats (Table 1).

**TABLE 1:** Levins' measure of niche breadth and Chi‐squared results for within‐species patterns for plant diet items, examining specifically within‐category variation, with each line being a single set of results.

	Bush rats			Possum		
	Levins	*χ* ^2^	*p*	Levins	*χ* ^2^	*p*
*Sex*						
Male	15.469	402.33	< 0.001	9.858	293.44	< 0.001
Female	13.210	190.27	< 0.001	9.283	150.75	< 0.001
NR	13.087	101.94	< 0.001	8.067	34.000	0.371
*Age*						
Adult	16.011	369.28	< 0.001	9.858	293.44	< 0.001
Subadult	15.781	82.921	< 0.001	6.368	46.000	0.052
Juvenile	7.562	74.000	< 0.001	—	—	—
NR	11.965	91.423	< 0.001	—	—	—
*Habitat*						
Casuarina	—	—	—	1.000	32.000	0.467
Forest	11.864	151.44	< 0.001	10.028	190.140	< 0.001
Heathland	10.314	41.789	0.115	—	—	—
Rainforest	13.158	75.400	< 0.001	4.765	53.333	0.010
Scrubland	12.000	42.000	0.111	—	74.857	< 0.001
Sedgeland	10.782	68.000	< 0.001	—	—	—
Shrubland	15.956	141.000	< 0.001	7.229	—	—
Woodland	12.261	133.62	< 0.001	5.556	49.400	0.025

*Note:* The Levins' measure is presented independently in the columns labelled ‘Levins’, and the one‐sample chi‐squared results are presented in the columns labelled ‘*χ* ^2’^ and ‘*p* value’. Dashes (—) represent categories where no samples were collected.

Overall, the Pianka index of overlap for the plant diets of bush rats and possums was 0.85. The test for dispersion demonstrated that the bush rat and possum datasets had a homogenous dispersion (*F*_1_ = 1.631, *p* = 0.204). However, PERMANOVA indicated the consumption of plant dietary items by bush rats and possums was significantly different (*F*_22_ = 21.224, *p* = 0.016). The species (i.e., bush rat or possum) accounted for 22.8% of the variance in the data. As the bipartite network illustrates, the largest amount of dietary overlap was in species from the orders Fabales (an order of flowering plants that includes peas, beans and soybeans) and Poales (grasses), both of which were the main plant sources for bush rats and possums (Figure 3B).

### 3.3 Invertebrate Diet Items

**FIGURE 4:**
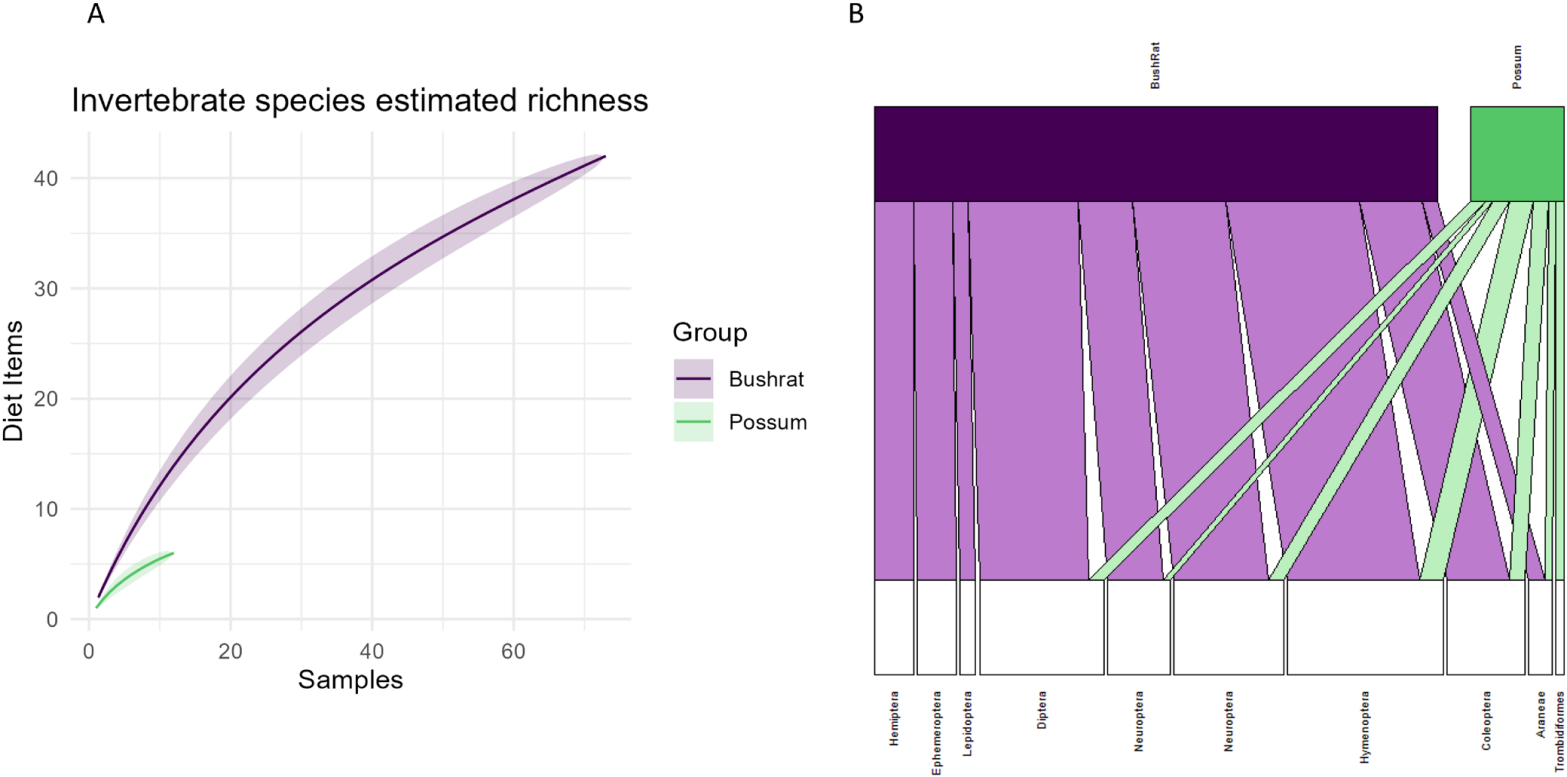
(A) Species accumulation curve of the accumulated number of diet items in the invertebrate taxa identified for bush rats and possums for the number of samples of each species, demonstrating that possums had a much smaller sample size, while also consuming very few invertebrate diet items. (B) Bipartite network plot of the invertebrate diet items detected in the faecal samples of bush rats and common brushtail possums at the ordinal level. This bipartite network illustrates which orders were consumed at higher frequencies, as well as illustrating where the overlaps in diets were occurring. In the invertebrate diet, most of the dietary overlap occurred in the Hymenoptera order.

We identified 24 invertebrate taxa from faecal samples; bush rats consumed 23 of these, and possums consumed seven (Figure 4A). The invertebrate taxa had the largest sample size difference between bush rats and possums, accounting for some of the observable differences in dietary breadth (Figure 4A). However, there was no statistical difference in the invertebrate dietary niche breadths of bush rats (11.02) and possums (5.54). Bush rats had an uneven frequency of occurrence of invertebrate dietary items ($\chi_{23}^{2}$  = 96.537, *p* < 0.001), mostly consuming invertebrates in the order Neuroptera (net‐winged insects). Possums also had an uneven frequency of occurrence of dietary items ($\chi_{23}^{2}$ = 40, *p* = 0.015), mostly consuming invertebrates in the orders Neuroptera and Hymenoptera (including bees, wasps, etc.) (Figure 4). Of diet items identified to species level, bush rats mostly consumed western black quill mayflies *Rhithrogena hageni* .

Within species, for both bush rats and possums, males and females had relatively similar invertebrate dietary niche breadths (Table 2). Male and female bush rats consumed some items more frequently than others (Table 2), with both more frequently consuming Neuroptera compared with other invertebrates. Male and female possums, conversely, had a more even spread in their dietary items (Table 2). Adult and subadult bush rats had greater dietary niche breadths than juvenile bush rats (Table 2). In comparison, only adult possums consumed invertebrates. Subadult and juvenile bush rats showed an even frequency of consumption. Adult bush rats and possums both consumed Neuroptera more frequently than other insect groups. The dietary niche breadths of individual bush rats and possums were relatively similar regardless of the habitat type in which they were trapped (Table 2). Additionally, with the exception of bush rats in shrubland habitats, there was an even frequency of consumption across the different habitat types (Table 2).

**TABLE 2:** Levins' measure of niche breadth and the Chi‐square results for the within‐species patterns for invertebrate diet items, examining specifically within‐category variation, with each line being a single set of results.

	Bush rats			Possum		
	Levins	*χ* ^2^	*p*	Levins	*χ* ^2^	*p*
*Sex*						
Male	11.215	61.556	< 0.001	5.538	40.000	0.015
Female	6.785	53.286	< 0.001	6.250	28.400	0.201
NR	8.522	50.857	0.001	INF	NA	NA
*Age*						
Adult	11.365	64.483	< 0.001	5.538	40.000	*P* = 0.015
Subadult	10.286	16.000	0.855	INF	NA	NA
Juvenile	5.000	19.000	0.701	—	—	—
NR	6.564	50.474	0.001	—	—	—
*Habitat*						
Casuarina	—	—	—	INF	NaN	NA
Forest	7.200	28.000	0.216	1.000	23.000	0.461
Heathland	4.500	26.000	0.301	—	—	—
Rainforest	8.758	29.588	0.162	1.000	23.000	0.461
Scrubland	5.000	19.000	0.701	—	—	—
Sedgeland	3.000	21.000	0.581	—	—	—
Shrubland	8.018	41.857	0.009	2.000	22.000	0.520
Woodland	8.100	35.333	0.048	3.000	21.000	0.581

*Note:* The Levins' measure is presented independently in the columns labelled ‘Levins’, and the one‐sample chi‐squared results are presented in the columns labelled ‘*χ* ^2^’ and ‘*p* value’. INF represents categories with too few samples to calculate the niche breadth and chi‐square. Dashes (—) represent categories where no samples were collected.

Overall, the Pianka index of overlap for the invertebrate diets of bush rats and possums was 0.74. The test for dispersion demonstrated that the bush rat and possum datasets had a homogenous dispersion (*F*_1_ = 2.086, *p* = 0.155). However, the invertebrate diets of bush rats and possums were significantly different (*F*_22_ = 1.212, *p* = 0.042). Species (i.e., bush rat or possum) accounted for 40.7% of the variance in the data. As the bipartite network illustrates, the greatest overlap in invertebrate diet was in items from the Hymenoptera order (Figure 4). The bipartite network additionally illustrated that bush rats consumed invertebrates from several orders that possums did not, while conversely, only possums consumed invertebrates from the order Trombidiformes (Figure 4B).

### 3.4 Fungi Diet Items

**FIGURE 5:**
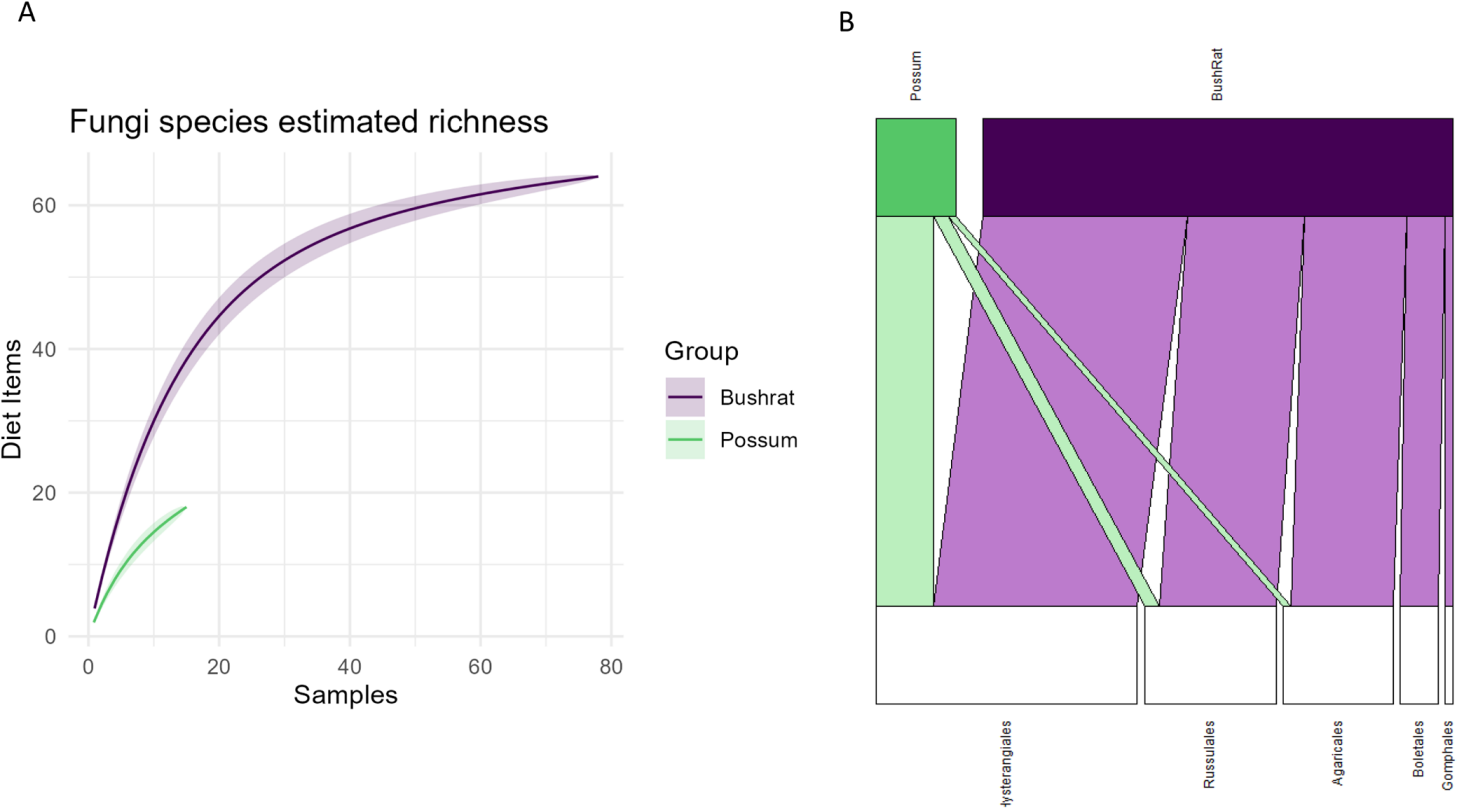
(A) Species accumulation curve of the accumulated number of diet items in the fungi taxa identified for bush rats and possums for the number of samples of each species, demonstrating the difference in the sample sizes between bush rats and possums, while also demonstrating bush rats consuming a wider range of fungi. (B) Bipartite network plot of the fungi diet items detected in the faecal samples of bush rats and common brushtail possums at the ordinal level. This bipartite network illustrates which orders were consumed at higher frequencies, as well as illustrating where the overlaps in diets were occurring. In the fungi diet, most consumption was in the Hysterangiales order, where most of the dietary overlap also occurred.

We identified 93 different ectomycorrhizal fungi from faecal samples; bush rats consumed 82 of these and possums consumed 12. This result can be observed in the species accumulation curve, which shows bush rats having a much greater dietary breadth across a much larger sample set (Figure 5A). There was no substantial difference in the dietary niche breadths of bush rats (9.984) and possums (3.045) (Figure 5). Both species showed differences in the frequency of occurrence of fungal items in the diet (bush rats: $\chi_{92}^{2}$  = 339.23, *p* < 0.001, possums: $\chi_{92}^{2}$  = 213.08, *p* < 0.001), with both mostly consuming fungal diet items from the order Hysterangiales (an order of truffles) (Figure 5).

Within species, for both bush rats and possums, males and females did not have substantially different dietary ranges (Table 3). Males and females of both species consumed some fungal items more than others, based on a chi‐squared analysis (Table 3), with all mostly consuming items from the genus *Hysterangium*. The dietary ranges of different age categories for both species were also not substantially different (Table 3). All age categories, except subadult possums which consumed items evenly, mostly consumed items from the genus *Hysterangium* (Table 3). Habitat types similarly did not reveal different dietary ranges for either bush rats or possums (Table 3). Most individuals in different habitat types consumed some items more than others, with all mostly consuming taxa in the genus *Hysterangium* (Table 3). The exception was possums in either woodland or rainforest habitats, which had an even frequency of consumption (Table 3).

**TABLE 3:** Levins' measure of niche breadth and the Chi‐square results for the within‐species patterns for fungal diet items, examining specifically within‐category variation, with each line being a single set of results.

	Bush rats			Possum		
	Levins	*χ* ^2^	*p*	Levins	*χ* ^2^	*p*
*Sex*						
Male	9.832	279.010	< 0.001	2.739	193.670	< 0.001
Female	8.379	126.440	< 0.001	2.964	109.770	< 0.001
NR	9.188	75.757	< 0.001	3.571	34.200	0.160
*Age*						
Adult	10.113	256.460	< 0.001	2.739	193.670	< 0.001
Subadult	7.364	75.667	< 0.001	3.000	25.000	0.574
Juvenile	6.533	46.000	0.013	—	—	—
NR	7.860	74.310	< 0.001	—	—	—
*Habitat*						
Casuarina	—	—	—	INF	NA	NA
Forest	8.225	137.040	< 0.001	2.945	153.110	< 0.001
Heathland	2.778	45.400	0.015	—	—	—
Rainforest	6.451	90.185	< 0.001	2.000	26.000	0.519
Scrubland	5.556	40.400	0.047	—	—	—
Sedgeland	4.765	43.889	0.021	—	—	—
Shrubland	9.887	78.767	< 0.001	1.800	43.677	0.022
Woodland	7.563	99.973	< 0.001	3.000	25.000	0.574

*Note:* The Levins' measure is presented independently in the columns labelled ‘Levins’, and the one‐sample Chi‐squared results are presented in the columns labelled ‘*χ* ^2^’ and ‘*p* value’. INF represents categories with too few samples to calculate the niche breadth and Chi‐square. Dashes (—) represent categories where no samples were collected.

Overall, the Pianka index of overlap of the fungal diets of bush rats and possums was 0.88. The dispersion test demonstrated that the bush rat and possum datasets had a homogeneous dispersion (*F*_1_ = 2.061, *p* = 0.155). Additionally, the fungal composition of bush rat and possum diets was not significantly different (*F*_19_ = 1.058, *p* = 0.276). Species (i.e., bush rats or possums) accounted for 24.5% of the variance in the data. As illustrated by the bipartite network, most of the overlap in diet arose from the consumption of fungi in the order Hysterangiales, which is the main dietary source of fungi for both bush rats and possums (Figure 5B).

## 4 Discussion

Our understanding of how bush rats and possums use and share resources is a vital step in assessing the likelihood of interspecific competition between these species during times of scarce resources (Wang et al. 2023). This study aimed to investigate the food resources used by bush rats and possums to determine a potential for competition by quantifying dietary overlap (Villsen et al. 2022).

Using DNA metabarcoding, we found that both species consumed a large range of food resources, indicative of dietary generalists, thus providing support for our first prediction that the diets of common brushtail possums and bush rats would encompass a broad range of food types. We found additionally that most of the items consumed by possums were also consumed by bush rats, thus supporting our second prediction that there would be a high overlap in the range of food types consumed by the two species. However, differences in diet were associated with bush rats having a much larger dietary range compared to possums, consuming many items that possums did not. Our findings provide some support for the proposition that competition could be a mechanism for the inverse numerical relationship between the two study species that we have observed in BNP (Kanishka et al. 2023). However, we found few fine‐scale differences between age and sex components of the populations and some habitat‐related differences in diet, refuting our third prediction that fine‐scale differences in diet would occur within and between species with respect to sex, age, and vegetation type where animals were active. Below, we explore the diets of the two species in more detail and also the likelihood and consequences of competition when it occurs between dietary generalists.

### 4.1 Diets of Bush Rats and Common Brushtail Possums

Both bush rats and common brushtail possums are considered to be omnivorous generalists (Cruz et al. 2012; Vernes et al. 2015), a result we corroborated only for bush rats in BNP. Possums and bush rats are widely distributed throughout Australia and commonly co‐occur in south‐eastern and south‐western coastal regions (Callander 2018; How and Hillcox 2000). Possums have additionally become an invasive species in New Zealand, where they consume varied plant species that do not occur in their natural range (Glen et al. 2012). The wide range of food types in the diet of possums, including plant material, invertebrates, anthropogenic foods and, occasionally, eggs and nestling birds (Glen et al. 2012; Gloury and Handasyde 2016; Herath et al. 2021; Scoleri et al. 2020), reflects the ability of possums to track diverse food resources on the ground and in trees (Cruz et al. 2012). It may also reflect the large differences in personality types and individual foraging preferences within local populations (Cruz et al. 2012; Herath et al. 2021). Bush rats consume many types of plants, invertebrates, and fungi (Vernes et al. 2015; Wanniarachchi et al. 2022). The species also shows dietary shifts in response to seasonal differences in the availability of different food types as well as environmental changes, such as those induced by fire (Vernes et al. 2015; Wanniarachchi et al. 2022). Dietary switching has previously been shown to allow the bush rat to maintain relatively stable populations by shifting from preferred to less‐preferred foods when preferred foods become scarce (Dickman and Happold 2022).

At BNP, most of the plants eaten by both species are found on the ground and in the understorey, particularly grasses like prickly couch grass, legumes, and other flowering plants. Given the time of sampling, in the late spring and through summer, these plant taxa were likely to be producing seeds and fruits that may be more preferred than leaves owing to their higher energy and nutrient contents (Morrant and Petit 2012), although we were unable to confirm which parts of the plants the species were consuming. Consumption of understorey plants was expected for bush rats, which are predominantly ground‐dwelling, but less so for possums, which spend a significant proportion of time moving and foraging in trees (Gloury and Handasyde 2016; How and Hillcox 2000). The increased ground‐level foraging by possums could reflect the availability of higher quality food on the ground and in the understorey, but could also be a result of lower predation risk due to the long‐term reduction of red fox activity by poison baiting at BNP (Dexter et al. 2013).

A notable difference in the diets of possums and bush rats was that, while most of the possum diet was plant material, a substantial proportion of the bush rat diet comprised truffle‐type fungi. Bush rats have previously been noted to be mycophagous, or consumers of fungi, so our results align with earlier studies (Vernes et al. 2015). Mountain brushtail possums *Trichosurus caninus* , a species closely related to common brushtail possums, have also been found to be mycophagous (Claridge and Lindenmayer 1998). Invertebrates comprised the smallest proportion of both species' diets, with possums consuming a near‐negligible amount. The latter result was unexpected, as a body of research and anecdotal observations note the omnivorous nature of possums (Cruz et al. 2012; Scoleri et al. 2023). Two main reasons could account for this result. First, possums in BNP may have a stronger preference for plant material, potentially due to a high density of palatable plants (Cruz et al. 2012; DeGabriel et al. 2009). Second, this result could be due to a failure to successfully extract invertebrate DNA from the faecal samples, resulting in a lower‐than‐actual representation of invertebrates in possum diets (Namin et al. 2022). The invertebrates we detected in the diets of our target species were mostly winged insects, particularly lacewings, mayflies, bees and wasps. Consumption of winged insects is surprising but may reflect the ingestion of larval rather than adult forms, as larvae would be easier for bush rats and possums to find in soil or under leaf litter. Alternatively, these could be a product of incidental consumption or environmental contamination, which may also account for some of the fungi diet results.

### 4.2 Limitations

There are several limitations to DNA metabarcoding which became apparent in this study and limit the conclusions and in‐depth investigation of both species' diets. First, many primers struggled to reach species‐level identification, limiting our species‐level comparisons of diet (Namin et al. 2022). A majority of the sequences identified in our study were identified at the order or genus level, while only a few were identified at the species level. While these results do provide a good overview of the types of dietary items that both species are consuming regularly (e.g., understorey plants), we do not know the precise degree of dietary overlap between bush rats and possums, as they may consume different species within the same genus (Villsen et al. 2022). Additionally, unlike histological and morphological methods, DNA metabarcoding can reveal only the identity of the dietary item but not information about what parts of that item are being consumed (Alemany et al. 2023). As mentioned above, while we can assume that both species consumed large amounts of flowering plants, based on the season when sampling took place, we cannot confirm if they consumed petals, leaves, roots, or other plant parts (Alemany et al. 2023). There is potential for misidentification of similar species; for example, *Acacia bellula* and *A. nematophylla* were both identified using the BLASTn tool, but neither has previously been found at or near Jervis Bay (Sritharan et al. 2021). This could be a result of a lack of reference data for many species, limiting the tools' ability to assign species effectively. Finally, we must account for the chance that some detections in both species may have resulted from incidental ingestion or even contamination during the collection and processing steps. This can be seen in the large number of non‐ectomycorrhizal fungi that were identified in the faecal samples.

### 4.3 Generalist–Generalist Competition

Given the substantial overlap in their diets, there is potential for competition to occur between bush rats and common brushtail possums (Wang et al. 2023) which, if confirmed, would be an example of generalist‐generalist competition (Barnes and Murphy 2023). According to Barnes and Murphy (2023), dietary generalist pairs are more likely to compete indirectly than directly, with the most generalist species – such as the moths *Malacosoma californicum* and *Hyphantria cunea* – in their study being temporally segregated. In Japan, the effect of competition from invasive raccoons *Procyon lotor* on native raccoon dogs *Nyctereutes procyonoides* was reduced because, while both species are generalist omnivores, they displayed different dietary preferences in habitats where they co‐occurred compared to where they foraged alone (Osaki et al. 2019). Osaki et al. (2019) noted that coexistence was helped by raccoon control programs that have kept raccoons at low density and that the situation may be different if raccoons increased and occupied all habitats where raccoon dogs were present. At BNP, possum detections have increased over many years due to the active management of red foxes in BNP (Kanishka et al. 2023). Although we cannot rule out the possibility that bush rats and possums encounter each other directly, this seems unlikely due to the rapid flight response of both species to auditory, visual or sometimes olfactory disturbance (Fardell et al. 2021, 2022; Warneke 1971). Instead, indirect interactions mediated by spatial and/or temporal avoidance are more likely, with the bush rat responding to the presence of the larger possum by diversifying the range of foods that it consumes.

Dietary generalist species often exhibit opportunistic feeding behaviours (de Carvalho et al. 2019; Osaki et al. 2019), consuming food types that may be only temporarily available or switching to less preferred foods if favoured foods are not available or are too costly to acquire. Following a severe fire, for example, Dickman and Happold (2022) found that bush rats increased their consumption of ferns and other less preferred foods that are available immediately post‐fire until preferred foods – dicotyledonous plants and fungi – had recovered. Opportunistic feeding can also be a product of avoiding competition (de Carvalho et al. 2019), as noted above in the case of raccoon dogs (Osaki et al. 2019). At BNP, we suggest that dietary opportunism provides a buffer for the bush rat to reduce the potential effects of competition from possums by providing exclusive access to a particularly diverse range of food types, and possibly also through diet switching. As diet switching can be confidently detected only by tracking changes in a forager's diet over time (or space) in relation to the available food resources, we suspect but cannot confirm the operation of diet switching in the bush rat at BNP.

Although there are very few areas within the broad geographical range of the bush rat where common brushtail possums do not also occur, we suggest it is unlikely that the dietary generalism of bush rats has evolved to reduce competition for food with possums. Bush rats have relatively small home ranges (< 1 ha), limited dispersal ability, and exhibit considerable flexibility in the range of microhabitats they inhabit (Ball et al. 2005; Fordyce et al. 2016; Reif et al. 2016). Dietary generalism in these circumstances may be selectively advantageous, with rats being able to shift between food resources that likely vary over time and with disturbances in their restricted home range areas (Fordyce et al. 2016; Peakall et al. 2006). Dietary generalist species often show considerable flexibility in habitat use and behaviours (Reif et al. 2016), as do many members of the genus *Rattus* (Aplin and Ford 2014). In fragmented habitats in Madagascar, a generalist folivore, the golden‐crowned sifaka *Propithecus tattersalli* , similarly demonstrated plasticity in its diet which allowed it to exploit remnant habitat patches and cope with limited dispersal opportunities (Quéméré et al. 2013). Dietary generalists are often likely to be more resilient to environmental disturbances such as fire or habitat fragmentation than dietary specialists (Dickman and Happold 2022; Quéméré et al. 2013). We, therefore, suggest that the ability to exploit varied food items also confers some resilience when the disturbance is in the form of a larger and dominant competitor.

### 4.4 Managing Food Competition

Bush rats are not threatened over their geographical range, but they experience localised population declines due to habitat loss and fragmentation, predation and, in BNP, potential competition with common brushtail possums. In protected areas where managers are charged with retaining populations of all indigenous species, how might dietary generalists such as the bush rat best be conserved? As the diverse diets of such species appear to provide a buffer against environmental disturbances and allow fine‐scale partitioning of food resources with dominant competitors (Adams et al. 2013; de Carvalho et al. 2019; Osaki et al. 2019), management should seek to retain the full spectrum of food resources. At BNP, management to reduce the adverse effects on bush rats of competition with common brushtail possums may be as simple as ensuring that plant diversity is retained or promoted (Băncilă et al. 2023; Casula et al. 2017; Vernes et al. 2015). The promotion of plant diversity should ensure that co‐dependent invertebrates and fungi are also supported, thus increasing available dietary items for bush rats and other dietary generalist species (Băncilă et al. 2023). Identifying the key food resources used and shared by at least the more common consumer species would be a fruitful research direction for managers of BNP and other protected areas.

## 5 Conclusions

Both bush rats and common brushtail possums are dietary generalists that show substantial overlap in their broad diets. This similarity in diets suggests that, in periods of restricted food availability, competition between bush rats and possums could have detrimental effects. However, both species are capable of utilising parts of the food resource base at finer scales of individual food types, and possibly also of switching diets to maximise the benefits of consuming such foods relative to the costs. In the case of the bush rat, a broad‐ranging diet likely provides a buffer against the negative impacts of competition from the larger brushtail possum, with individuals that may be more susceptible to competition reducing their risk of encounters with possums by constraining their dietary range and foraging in safer habitats. Our results demonstrate that even when there is a high similarity in the foods consumed by dietary generalist species, fine‐scale partitioning of food types within and between competitor populations, or possibly diet switching, of food resources may be important in alleviating competition and permitting co‐existence.

## Author Contributions

**Aurelie M. Kanishka:** conceptualization (lead), data curation (lead), formal analysis (equal), funding acquisition (lead), investigation (lead), methodology (lead), project administration (lead), visualization (lead), writing – original draft (lead). **Christopher MacGregor:** conceptualization (supporting), data curation (supporting), methodology (supporting), resources (supporting), validation (equal), writing – review and editing (equal). **Linda E. Neaves:** conceptualization (supporting), data curation (supporting), formal analysis (equal), methodology (supporting), resources (equal), validation (equal), writing – review and editing (equal). **Maldwyn John Evans:** conceptualization (supporting), formal analysis (equal), methodology (supporting), software (lead), supervision (supporting), validation (equal), visualization (supporting), writing – review and editing (equal). **Natasha M. Robinson:** conceptualization (supporting), supervision (supporting), validation (equal), writing – review and editing (equal). **Nick Dexter:** conceptualization (supporting), supervision (supporting), validation (equal), writing – review and editing (equal). **Chris R. Dickman:** conceptualization (supporting), supervision (supporting), validation (equal), writing – review and editing (equal). **David B. Lindenmayer:** conceptualization (supporting), project administration (supporting), resources (equal), supervision (lead), validation (equal), writing – review and editing (equal).

## Conflicts of Interest

The authors declare no conflicts of interest.

## Supporting information


Appendix S1.

## Data Availability

The data that support the findings will be made publicly available on the FigShare data repository upon acceptance of this manuscript (reviewer link: https://doi.org/10.6084/m9.figshare.26095246, https://doi.org/10.6084/m9.figshare.26103502).
