# Supplementary material for: Quantifying the Dietary Overlap of Two Co‐Occurring Mammal Species Using DNA Metabarcoding to Assess Potential Competition
Source: Ecol Evol. 2025 Apr 11;15(4):e71274. doi: 10.1002/ece3.71274 (PMC11992362; doi:10.1002/ece3.71274)
Supplement: Supplementary file 1 — Appendix S1. [file ECE3-15-e71274-s001.docx]

**Supplementary Material**

Table S1: The number of samples collected by species, age, sex and vegetation type.

| **Bush Rat** | | **Possum** | |
| --- | --- | --- | --- |
| Total | 78 |  | 57 |
| *Period* | | | |
| First | 20 | First | 20 |
| Second | 58 | Second | 37 |
| *Sex* | | | |
| Male | 27 | Male | 23 |
| Female | 39 | Female | 31 |
| Not Recorded | 12 | Not Recorded | 3 |
| *Age* | | | |
| Adult | 46 | Adult | 50 |
| Juvenile | 9 | Juvenile | 1 |
| Subadult | 14 | Subadult | 3 |
| Not Recorded | 9 | Not Recorded | 3 |
| *Habitat* | | | |
| Casuarina | 0 | Casuarina | 4 |
| Forest | 20 | Forest | 32 |
| Heathland | 4 | Heathland | 2 |
| Rainforest | 11 | Rainforest | 4 |
| Scrubland | 3 | Scrubland | 1 |
| Sedgeland | 6 | Sedgeland | 0 |
| Shrubland | 19 | Shrubland | 8 |
| Woodland | 15 | Woodland | 6 |

Table S2: The list of identified diet items, separated by plant, invertebrate, and fungi, detailing the level of resolution we were able to identify diet items to (i.e., species, genus, order).

| **Plant** | | **Invertebrate** | | **Fungi** | |
| --- | --- | --- | --- | --- | --- |
| **Species** | **Level** | **Species** | **Level** | **Species** | **Level** |
| *Avena* | genus | Arthropoda | phylum | *Hysterangium* | genus |
| Fabaceae | family | *Rhithrogena hageni* | species | *Russula vinaceocuticulata* | species |
| Poales | order | Neuroptera | order | *Hysterangium pterosporum* | species |
| *Acacia* | genus | Sciaridae | family | *Chondrogaster pachysporus* | species |
| *Schelhammera undulata* | species | *Pheidole* | genus | *Mesophellia oleifera* | species |
| Proteaceae | family | Coleoptera | order | *Cortinarius* | genus |
| Orchidaceae | family | Hymenoptera | order | *Scleroderma* | genus |
| Poaceae | family | Diptera | order | *Russula floriformis* | species |
| *Bossiaea* | genus | *Anonychomyrma* | genus | *Protoglossum niphophilum* | species |
| Apiaceae | family | Phoridae | family | *Mesophellia* | genus |
| *Patersonia glabrata* | species | Araneae | order | *Thaxterogaster dulciorum* | species |
| Pittosporaceae | family | *Olios* | genus | *Russula* | genus |
| *Pinus* | genus | *Symplecta pilipes* | species | *Timgrovea* | genus |
| Rutaceae | family | Hemiptera | order | *Russula purpureoflava* | species |
| Oleaceae | family | *Solenopsis* | genus | *Cortinarius clelandii* | species |
| Rubiaceae | family | *Apis mellifera* | species | *Russula foetens* | species |
| *Dracaena* | genus | Cecidomyiidae | family | *Austrogautieria* | genus |
| *Glycine* | genus | *Diplonevra* | genus | *Hallingea* | genus |
| *Elaeocarpus* | genus | *Camponotus intrepidus* | species | *Chondrogaster* | genus |
| *Eustrephus latifolius* | species | Chloropidae | family | *Malajczukia amicorum* | species |
| *Viola* | genus | *Diarsia intermixta* | species | *Cortinarius beeverorum* | species |
| *Zoysia macrantha subsp. walshii* | species | *Persectania ewingii* | species | *Gymnomyces* | genus |
| *Asparagus aethiopicus* | species | *Idiodes apicata* | species | *Russula sinuata* | species |
| *Liparophyllum exaltatum* | species | Tetranychidae | family | *Arcangeliella claridgei* | species |
| *Acacia nematophylla* | species | *Ixodes holocyclus* | species | *Gyroporus* | genus |
| *Crassula* | genus | Braconidae | family | *Octaviania tasmanica* | species |
| *Casuarina* | genus | *Mimaglossa nauplialis* | species | *Inocybe bulbinella* | species |
| *Smilax* | genus | *Sphaleractis eurysema* | species | *Fistulinella* | genus |
| Myrtaceae | family |  |  |  |  |
| *Spiranthes sinensis var. amoena* | species |  |  |  |  |
| Asparagales | family |  |  |  |  |
| *Acacia bellula* | species |  |  |  |  |
| *Cinnagrostis viridis* | species |  |  |  |  |
| Asteraceae | family |  |  |  |  |
| Caryophyllales | order |  |  |  |  |
| *Eucalyptus vernicosa* | species |  |  |  |  |
